# Ventromedial Prefrontal Cortex Activation Is Associated with Memory Formation for Predictable Rewards

**DOI:** 10.1371/journal.pone.0016695

**Published:** 2011-02-04

**Authors:** Katharina A. Bialleck, Hans-Peter Schaal, Thorsten A. Kranz, Juergen Fell, Christian E. Elger, Nikolai Axmacher

**Affiliations:** 1 Department of Epileptology, University of Bonn, Bonn, Germany; 2 Life and Brain Center for Academic Research, Bonn, Germany; University of Minnesota, United States of America

## Abstract

During reinforcement learning, dopamine release shifts from the moment of reward consumption to the time point when the reward can be predicted. Previous studies provide consistent evidence that reward-predicting cues enhance long-term memory (LTM) formation of these items via dopaminergic projections to the ventral striatum. However, it is less clear whether memory for items that do not precede a reward but are directly associated with reward consumption is also facilitated. Here, we investigated this question in an fMRI paradigm in which LTM for reward-predicting and neutral cues was compared to LTM for items presented during consumption of reliably predictable as compared to less predictable rewards. We observed activation of the ventral striatum and enhanced memory formation during reward anticipation. During processing of less predictable as compared to reliably predictable rewards, the ventral striatum was activated as well, but items associated with less predictable outcomes were remembered worse than items associated with reliably predictable outcomes. Processing of reliably predictable rewards activated the ventromedial prefrontal cortex (vmPFC), and vmPFC BOLD responses were associated with successful memory formation of these items. Taken together, these findings show that consumption of reliably predictable rewards facilitates LTM formation and is associated with activation of the vmPFC.

## Introduction

Only a small fraction of all sensory information available in a given moment is encoded into long-term memory (LTM). One important criterion for LTM encoding of a particular stimulus is its relationship to rewards. In general, stimuli which are either themselves rewarding or reliably predict reward in the near future are salient information which should be remembered to guide future behavior. These general laws hold for many species, and the neurobiological basis linking reward processing and memory formation has been investigated with both electrophysiological and neuroimaging methods. Activation of dopaminergic midbrain neurons is highly rewarding, and rodents with stimulation electrodes in this region attempt to receive stimulation [1]. More recently, electrophysiological recordings in monkeys showed that when a particular cue invariantly predicts an upcoming reward, midbrain neurons already increase their firing rate upon presentation of the cue, but consumption of the reward itself does not affect firing rate additionally [2]. Moreover, while neurons in the ventral striatum – and in particular the nucleus accumbens – showed neural activity during reward anticipation, the orbitofrontal cortex (OFC) was active during actual reward consumption [3]. Less clear results were obtained in fMRI studies in humans. While some researchers found a dissociation between activity during reward anticipation in the ventral striatum and reward consumption in the ventromedial prefrontal cortex (vmPFC) including the OFC (e.g., [4], [5]), others observed that the ventral striatum was also activated during reward outcome [6]–[8].

In rodents, release of dopamine in structures of the medial temporal lobe (MTL) that are crucial for LTM encoding facilitates synaptic plasticity [9], [10]. Similarly, application of dopaminergic drugs in patients with Parkinson's disease (showing reduced levels of dopamine release) improves their memory performance, e.g. [11]. FMRI studies in healthy subjects showed that presentation of items predicting a reward induces an increased BOLD response in the hippocampus and is associated with enhanced memory for those items [12]. Moreover, memory for items presented during a task which is followed by a reward in case of successful completion is also enhanced if the reward can be predicted [13].

Taken together, these studies provide converging evidence for facilitated LTM encoding of cues which predict an upcoming reward via enhanced dopaminergic activation of the MTL. It is less clear, however, whether items which do not precede a predicted reward but are directly associated with consumption of a predicted reward are also better encoded into LTM. As the firing rate of dopaminergic neurons may not be affected by these stimuli anymore [14], memory for predicted rewards might not differ from memory for neutral stimuli. On the other hand, it may be argued that reward consumption in these situations remains highly salient and thus needs to be memorized. Here, we investigated these competing hypotheses. We scanned subjects via fMRI using a modified version of the experimental design by Wittmann and colleagues [12], in which recognition memory for reward-predicting and neutral cues was compared. In addition, we tested memory for items presented during reward consumption, which could be either reliably predicted by a cue or not. Items presented at the position of reward consumption were labeled ‘outcomes’ and contrasted to ‘cues’, i.e. items predicting a reward. Note that only about half of the outcomes were associated with an actual monetary reward, which was signaled by a green frame around the outcomes, while the rest was associated with a red frame around the outcome, indicating that subjects did not receive a reward in this trial.

## Methods

### Ethics statement

The study was approved by the local medical ethics committee (“Ethikkommission an der Medizinischen Fakultaet der Rheinischen Friedrich-Wilhelms-Universitaet Bonn”), was according to the latest version of the Declaration of Helsinki, and all subjects provided written informed consent.

### Subjects

Twenty healthy adults who were recruited from the University of Bonn participated in the study, five of which were excluded afterwards due to excessive motion artifacts. Of the remaining 15 subjects (8 female), mean age (± std.) was 23.87 (±1.92) years.

### Experimental paradigm

#### Overview

**Figure 1:**
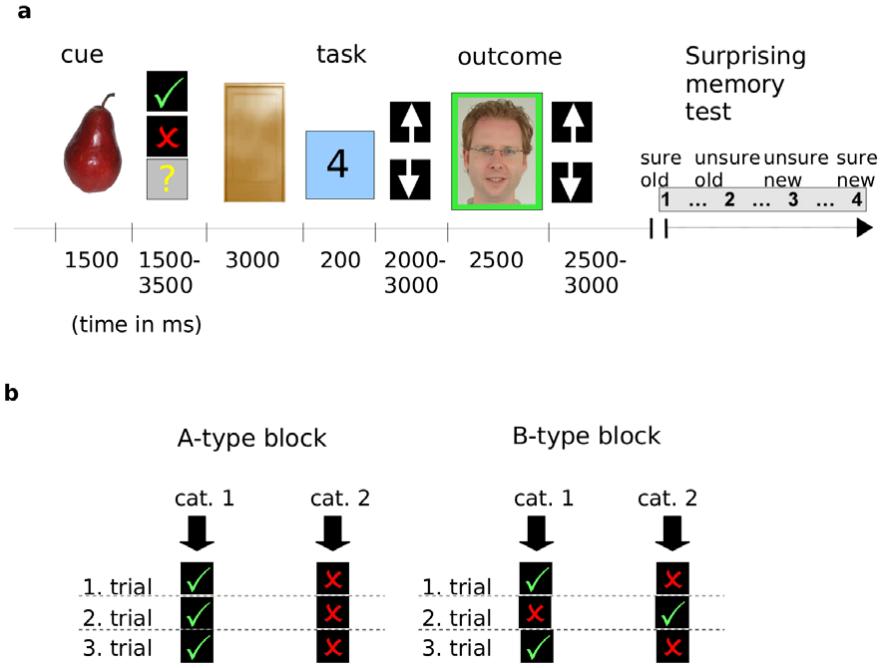
Overview of the experimental paradigm. (a) During scanning, subjects engaged in a number comparison task, which was followed by a monetary reward in some trials if correctly solved, while other trials were not rewarded. Prior to this task, a trial-unique cue from one of two categories was presented. Subjects had to infer from their experiences whether they were in an A-type block where the category of a cue predicted reward or not or in a B-type block, where cue category did not predict anything. During the outcome phase, a face surrounded by a green frame signaled a reward, while faces with red frames signaled no reward. After completion of 10 blocks and a break of 30 min, a surprise recognition memory task for all items presented as cues and during the outcome phase followed. (b) Schematic overview of trials within predictable and unpredictable blocks. Within predictable A-type blocks, the category of cue items reliably predicted the possibility of a reward after the number comparison task. In unpredictable B-type blocks, such a prediction was not possible, and rewards were thus received unpredictedly.

We used an event-related fMRI design in a reward anticipation paradigm with a subsequent recognition memory test [12]. A schematic depiction of the paradigm is presented in Fig. 1A. Prior to scanning, participants were given detailed information about the task and completed a practice version consisting of two blocks.

During the experiment, subjects completed ten blocks (lasting about 5 min each). These were divided into 5 predictable blocks (A-type blocks) and 5 unpredictable blocks (B-type blocks) in random order. In A-type blocks, subjects could predict the possibility of a reward by the category of the stimulus which was presented as cue, whereas such a prediction was not possible in B-type blocks. In both blocks, each trial included an anticipation phase, during which subjects indicated whether they expected a reward or not, a task-phase consisting of a number comparison task, and an outcome phase informing subjects about the monetary outcome of the particular trial. Cues consisted of photographs of living and nonliving objects collected from the internet, and were from two different categories in each block (e.g., clothes and pets, garden tools and home appliances, etc.). Participants did not know these categories in advance, but had to infer the classifications in each block: They had to learn to which two categories the presented cues belonged. In A-type blocks, pictures from one category signaled that the upcoming number comparison task would be rewarded if solved correctly and fast enough, the other one predicted that no reward would be provided even if the task was solved correctly. In B-type blocks also half of the trials were rewarded if the number comparison task was completed successfully, but this was not predicted by the pictorial cues. Instead, rewards were randomly distributed. For each subject, categories were randomly assigned to the class of rewarding or non-rewarding cues in A-type blocks or to B-type blocks.

#### Learning during the blocks

At the beginning of each block, participants did not know whether they were in an A-type block or in a B-type block. This had to be inferred by the presence (A-type blocks) or absence (B-type blocks) of a fixed relationship between stimulus categories and obtained rewards. In other words, subjects had to conclude from the reward outcomes during the block whether there was such a predictable system and how the categories were defined (A-type blocks) or whether there was no such structure at all but rewards were randomly distributed (B-type blocks; Fig. 1B).

Subjects were explicitly informed that, in predictable A-type blocks, cues were 100% predictive (as long as the task was performed correctly and sufficiently rapid). This is reflected by the fact that they learned the rules very fast during the blocks – on average, after the first 4±0.72 items, which is close to the theoretical limit. As criterion for learning of reward contingencies, we selected the first correct response which was followed by at least two other correct responses and no more than a single incorrect response in a row in the rest of the block).

#### Experimental details

Each block consisted of 20 trials with an average duration of about 15 s each, and subjects received either 0.40 € per trial or nothing. In each block, ten trials were potentially rewarded if the number comparison task was solved correctly and fast enough (see below), the others were not rewarded regardless of task performance. The order of rewarding and non-rewarding trials was random in each block, with the exception that there were not more than 3 items of one category in a row. Each trial started with a cue picture for 1500 ms. Subjects were required to press a button with the right index if they expected a reward in logical A-type blocks; with the left index if they expected no reward in A-type blocks; and with the right thumb if they believed to be in an illogical B-type block. This classification of stimulus category was implemented to ensure that subjects learned the reward contingencies; incorrect classifications did not influence the outcome of the trial. Although this was thoroughly explained to each subject, some subjects in piloting experiments still expressed their belief during debriefing that there was such an influence. To avoid this assumption, which may interfere with processing of the relationship between cues and rewards, subjects were instructed to imagine that they participated in a game show where cues were attached on the front of doors to indicate possible rewards which could be obtained in a game (the number comparison task) played in the room behind the doors. This familiar imagination implied that the relationship between cues and rewards did not depend on the subjects' estimation about the meaning of a cue. To facilitate this imagination, after a variable interval of 1500–3500 ms after the cue a brief video sequence of an opening door (3000 ms) announced the number comparison task. As soon as the door was open, subjects indicated as fast as possible whether a number with random values from 1 to 9 (except 5), which was presented for 200 ms, was greater than 5 by pressing the right button, or smaller than 5 by pressing the left button. This task had to be performed during an individually adjusted response deadline which depended on the reaction times in the five immediately preceding trials such that the correct response rate was 80%. In the first five trials of each block, where no preceding trials could be used for this calculation, winning or losing was randomized (with an average rate of 80% win trials for correct solutions to the number comparison task after a cue of the win category in A-type blocks). Following a variable delay of 2000–3000 ms after the response, subjects received visual feedback in form of a male or female face with neutral emotional expression which was presented for 2500 ms. Pictures were analyzed by the authors for emotional expression, and only faces rated as neutral were used for the experiment. Faces were surrounded by a green frame to signal a reward (satisfactory performance in rewarding trials); by a grey frame to indicate no reward despite good performance (satisfactory performance in non-rewarding trials); and by a red frame to signal unsatisfactory performance in both conditions. Face stimuli were randomly assigned to the different conditions in each subject. Finally, a variable fixation phase of 2500–3500 ms followed prior to the next trial. Because during piloting, subjects paid little attention to the faces, but only to their colored frame (reward/no reward), we added the additional question whether the person was older or younger than 30 years, to draw subjects' attention to the faces. Subjects had to press the right button if they considered the person to be older and the left button, if younger.

#### Instructions and recognition memory test

Participants were asked to pay attention to the cues in order to infer from the outcome whether they were in A-type blocks or in B-type blocks, but they were not informed about the subsequent recognition memory test. Thirty minutes after scanning, subjects completed a surprise recognition memory test (outside of the scanner). All pictures presented as cues and all faces presented during reward consumption plus 50% new items of both categories were presented on a computer screen. Memory for each items was rated on a four-point scale (1 =  sure old; 2 =  unsure old; 3 =  unsure new; 4 =  sure new). Timing of presentation was self-paced. Due to the relatively large number of experimental conditions (predictability, reward, item type, subsequent memory), the number of ‘sure old’ and ‘sure new’ responses in each condition was insufficient for an analysis of fMRI data: If only ‘sure old’ and ‘sure new’ responses were taken into account, there was not a single subject with at least 10 trials in each condition. Therefore, we collapsed across trials with ‘sure’ and ‘unsure’ responses. We are aware, however, that an influential theory [15] suggests that memory for items with subsequent ‘sure old’ responses is based on a different process (recollection) than for items with subsequent ‘unsure old’ responses (familiarity). Also, different neural substrates have been suggested for these two memory processes: The anterior hippocampus for recollection, and the rhinal cortex for familiarity. This is described in greater detail in the Discussion section.

Because participants were unable to differentiate between predictable and unpredictable blocks during the first three trials in each block, we removed data of these trials in each block. Hence our analysis included a total of 340 pictures per participant: Eighty-five cue pictures predicting a possible reward or predicting no reward (in A-type blocks), 85 cues in B-type blocks, and 170 faces presented during reward consumption. The exact number of rewarding outcomes differed according to individual performance. On average, participants were presented 34.4 (s.e.m.: ±1.1) rewarding and 50.7 (±1.1) non-rewarding outcomes in predictable blocks; and 33.1 (±0.8) rewarding and 51.9 (±0.8) non-rewarding outcomes in non predictable blocks. The paradigm was presented using Presentation software (version 0.71, www.neurobs.com), and all stimuli were presented using video goggles.

### FMRI recordings

Thirty-six axial slices were collected at 3T (Trio, Siemens, Erlangen, Germany). We collected 1250 T2*-weighted, gradient echo EPI scans (slice thickness: 2.0 mm; inter-slice gap: 1.0 mm; matrix size: 128×128; field of view: 230×230 mm; echo time: 33 ms; repetition time: 2700 ms). Thereafter, we acquired a 3D-sagittal T1-weighted MPRAGE sequence for each subject for anatomical localization (number of slices: 160; slice thickness: 1 mm; inter-slice gap: 0.5 mm; voxel size: 1×1×1; matrix size 256×256; field of view: 256 mm; echo time: 3.42 ms; repetition time: 1570 ms).

Preprocessing was done using FSL software (FMRIB's Software Library, www.fmrib.ox.ac.uk/fsl) and the following steps were performed: (1) Realignment with three-dimensional motion correction. (2) Normalization onto the MNI-atlas (Montreal Neurological Institute). (3) Spatial smoothing with an 8 mm Gaussian kernel (full width at half maximum). (4) Modeling of the expected hemodynamic responses (box-car regressor in a general linear model, GLM) and convolution of the regressors with a canonical hemodynamic response function to represent brain physiology. We used regressors using delta pulses triggered to the presentation of each stimulus. The following set of 16 regressors was used: Eight regressors for the predictable block, four for the cues and four for the outcomes. The four cues were (a) the reward-predicting, later remembered ones, (b) the reward-predicting, later forgotten ones, (c) the no-reward-predicting, later remembered ones, (d) the no-reward-predicting, later forgotten ones. The four outcomes were (a) the rewarding, later remembered ones, (b) the rewarding, later forgotten ones, (c) the not rewarding, later remembered ones, (d) the not rewarding, later forgotten ones. The other eight regressors were used accordingly for the unpredictable block, also four for the cues and four for the outcomes. Next (5), data were temporally filtered to reduce high- and low- frequency noise attributable to scanner drifts and physiological noise. The subsequent steps were conducted using SPM2 (www.fil.ion.ucl.ac.uk/spm/): (6) Calculation of parameter estimates for each condition covariate from the least mean squares fit of the model to the data. (7) Random-effects group analyses with subject as the random factor were performed with SPM2 on each regressor by entering the t-contrast images of each subject corresponding to a particular regressor into a second-level one-sample t-test. (8) Definition of contrasts (described in detail in the Results section).

Figures with fMRI results are displayed using neurological convention (left hemisphere on the left side of the figure). To identify significant activations, we used an uncorrected threshold of p<0.001 and a minimum cluster size of 5 contiguous voxels unless indicated otherwise. As we were specifically interested in activation differences in the MTL (hippocampus and parahippocampal cortex), ventral striatum, and vmPFC, we masked all contrasts with an anatomically pre-defined mask including these three regions. Using this mask, our a priori search volume is substantially smaller than the entire brain, and thus the risk of false positive activations when using a threshold of p<0.001 and 5 voxels is reduced proportionally (for a similar procedure, see [16]). Statistical analyses of behavioral data were performed using SPSS (SPSS Inc., Chicago, Illinois).

## Results

### Behavioral data

Participants received monetary rewards on an average of 79.6% (±1.3%) of all trials, thereof significantly more money in predictable tasks (F_1,14_ = 6.753; p = 0.021). The average rate of all correct and sufficiently rapid responses over all trials was 76.9% (±0.8%), approximating the maximum 80% correct response rate.

#### Reaction times and accuracy rates

**Figure 2:**
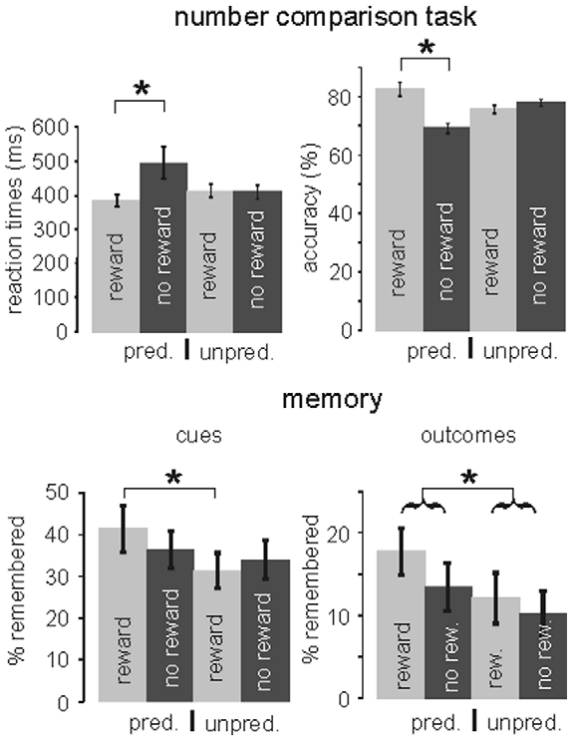
Behavioral results. Top: Performance in the number comparison task. Left: In predictable blocks, RTs were significantly shorter in rewarded as compared to non-rewarded trials. Right: Similarly, accuracy was significantly lower in predictably not rewarded trials as compared to predictably rewarded trials. Bottom: Memory for items presented as cues and outcomes. Left: Memory was significantly better for cues which predicted a reward as compared to cues which were unpredictedly followed by a reward. Right: Items presented during processing of predictable outcomes were remembered better than those during unpredictable outcomes. Pred.  =  predictable, unpred.  =  unpredictable, rew.  =  reward, no rew.  =  no reward.

First, we analyzed reaction times (RTs) and response accuracy in the number comparison task (Fig. 2). A two-way ANOVA of RTs with the repeated measures ‘predictability’ (predictable A-type vs. unpredictable B-type blocks) and ‘reward’ (rewarding vs. non rewarding trials in each block) revealed a significant effect of ‘reward’ (F_1,14_ = 8.637; p = 0.011) and a significant interaction (F_1,14_ = 6.997; p = 0.019). In predictable A-type blocks, reaction times in rewarded trials were significantly shorter than in non-rewarded trials (384.0 ms ±17.4 ms vs. 495.1 ms ±47.1 ms; F_1,14_ = 7.949; p = 0.014). In unpredictable B-type blocks, there was no difference between reaction times (413.2 ms ±20.6 ms vs. 409.2 ms ±19.9 ms; F_1,14_ = 0.724; p = 0.609).

For accuracy, a two-way ANOVA with the repeated measures ‘predictability’ and ‘reward’ revealed a significant effect of ‘reward’ (F_1,14_ = 9.6; p = 0.008), and a significant ‘predictability’ × ‘reward’ interaction (F_1,14_ = 19.521; p = 0.001). Within predictable blocks, significantly more correct responses were given in rewarded (83.07% ±2.3%) than in non-rewarded trials (69.73% ±1.61; F_1,14_ = 16.451; p = 0.001), while in unpredictable blocks there was no difference between rewarding and non-rewarding trials. Taken together, subjects performed faster and more accurate in rewarding trials of predictable A-type blocks as compared to non-rewarding trials in these blocks and to unpredictable trials from B-type blocks.

#### Memory effects

Next, we analyzed memory performance for the stimuli presented as cues and outcomes. Figure 2 (bottom) shows the percentage of hits (collapsed across “sure old” and “unsure old”) minus the percentage of false alarms (again collapsed across “sure old” and “unsure old”). In total, 200 cues and 200 faces were presented during the encoding phase, and an additional 50% during the retrieval phase (i.e., 200 old cues, 100 new cues, 200 old faces, and 100 new faces during retrieval). As noted above, only about half of the cues predicted a reward, and about half of the outcomes were associated with actual receipt of a reward. A three-way ANOVA with ‘predictability’, ‘item type’ and ‘reward’ as repeated measures revealed main effects of ‘predictability’ (F_1,14_ = 10.271; p = 0.006), indicating better memory for cues and outcomes in predictable blocks, and ‘item type’ (F_1,14_ = 1.501; p<10^−4^), indicating that cues were better remembered than outcome stimuli. A trend for a ‘predictability’ × ‘reward’ interaction (F_1,14_ = 3.314; p = 0.09) may reflect different reward effects on memory in predictable and unpredictable blocks. Because we were interested in differential effects during reward anticipation and consumption, we conducted separate analyses for stimuli presented as cues and outcomes.

For cues, there was a significant difference with respect to memory performance between reward-predicting cues in predictable A-type blocks as compared to cues from rewarded trials in unpredictable B-type blocks (F_1,14_ = 5.772; p = 0.031), while memory for the non-rewarding cues in the two conditions was not different (F_1,14_ = 0.780; p = 0.392). A similar effect was observed for outcomes: Again, participants remembered significantly more stimuli presented in predictable A-type blocks as compared to unpredictable B-type blocks (F_1,14_ = 5.577; p = 0.033). In addition, there was a trend for an effect of “reward” (F_1,14_ = 3.721; p = 0.074), but no ‘predictability’ × ‘reward’ interaction (F_1,14_ = 0.325; p = 0.578). Finally, there was a trend for better memory of rewarded outcomes in predictable as compared to unpredictable blocks (F_1,14_ = 3.315; p = 0.090). Thus, both cues and outcomes were better remembered in a predictable situation; for cues, this effect was specifically due to increased memory for reward-predicting cues.

Finally, we analyzed memory only with regard to “sure old” responses. For cues, this analysis revealed that reward-predicting cues in predictable A-type blocks were remembered better than cues from rewarded trials in unpredictable B-type blocks (F_1,14_ = 6.478; p = 0.023). This effect was absent for non-rewarded trials (F_1,14_ = 0.008; p = 0.932). In addition, in predictable blocks reward-predicting cues were better remembered than cues predicting no reward (F_1,14_ = 6.428; p = 0.024); in unpredictable blocks, no such effect became apparent (there was even a trend for a better memory of stimuli which were not followed by a reward; F_1,14_ = 3.340; p = 0.089). For outcomes, there was a main effect of “predictability” indicating better memory in the predictable condition (F_1,14_ = 10.569; p = 0.006), but no effect of “reward” and no interaction; however, we also observed a trend for better memory to outcomes presented during predicted as compared to unpredicted rewards (F_1,14_ = 3.109; p = 0.090).

### FMRI Results

We will first describe BOLD responses associated with cues and outcomes separately, and then compare the two. It should be noted that differences in activation during processing of cues and outcomes might be related to the fact that different stimulus categories were used during these two experiment stages (objects as cues, faces as outcomes); this is discussed in greater detail below.

#### Cues

**Figure 3:**
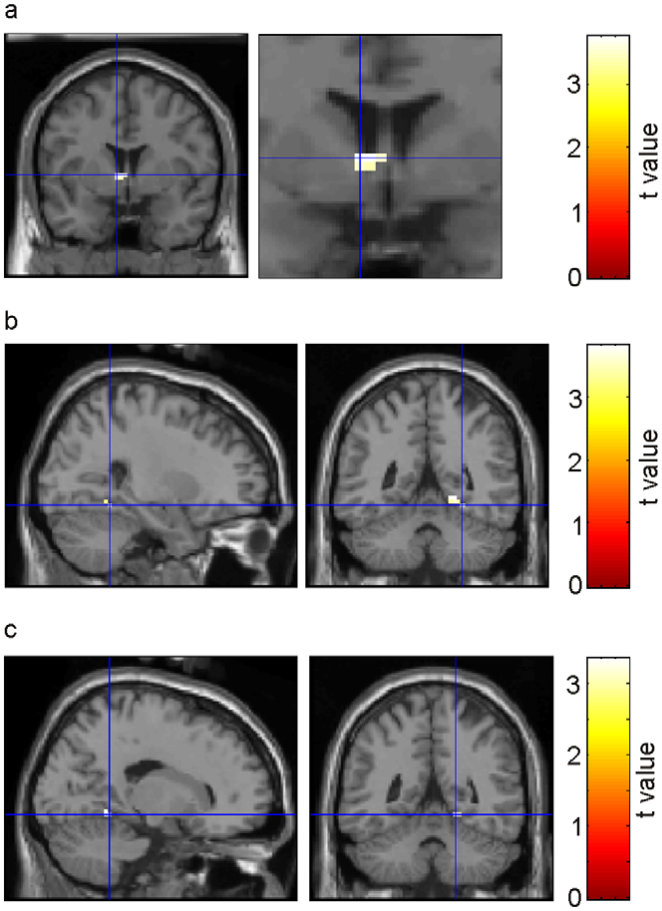
Activation of ventral striatum and parahippocampal cortex during reward anticipation facilitates memory formation. (a) Increased BOLD response in the ventral striatum for reward-predicting as compared to no reward predicting cues. (b) Increased BOLD response in the right parahippocampal gyrus for remembered contrasted to forgotten predicting cues. (c) Enhanced BOLD activity in the right parahippocampal gyrus associated with the interaction of ‘precitability’ × ‘memory’.

First, we aimed at testing the previous finding that reward-predicting items activated the ventral striatum to a stronger degree than items predicting no reward [12]. We thus contrasted reward-predicting cues to no-reward-predicting cues in predictable blocks. Indeed, this analysis revealed enhanced activity in the left ventral striatum (caudate nucleus; peak MNI coordinates: -6, 3, 3; Fig. 3A; see table 1 for an overview of all significant clusters of activation). These activations may underlie the effects of reward on accuracy and RTs in the number comparison task in predictable blocks.

**Table 1:** Overview of all significant clusters of activation in all relevant contrasts.

				MNI coordinates		
k_E_	t value	p_uncorrected_	x	y	z	structure
**Cues in predictable blocks: reward-predicting vs. no reward predicting (** **Fig. 3A** **)**						
10	3,74	0.0002	-6	3	3	caudate nucleus
**Cues in predictable blocks: remembered vs. forgotten (** **Fig. 3B** **)**						
18	3,82	0.00008	21	-51	-6	parahippocampal gyrus
	3,33	0.00051	27	-48	-12	parahippocampal gyrus
**Cues: 'predictability' × 'memory' interaction (** **Fig. 3C** **)**						
5	3,35	0.00047	18	-48	-9	parahippocampal gyrus
**Outcomes: predicted rewards vs. unpredicted rewards (** **Fig. 4A** **)**						
49	4,14	0.00002	-3	39	-15	medial orbitofrontal cortex
**Outcomes: unpredicted rewards vs. predicted rewards (** **Fig. 4B** **)**						
26	4,42	0.000007	-12	12	6	caudate nucleus
13	3,86	0.00006	15	9	0	internal capsule
**Outcomes: predicted vs. unpredicted (** **Fig. 4C** **)**						
45	3,70	0.0001	0	45	-15	medial orbitofrontal cortex
	3,53	0.0003	0	36	-9	vmPFC
**Outcomes: unpredicted vs. predicted (** **Fig. 4D** **)**						
14	4,02	0.00004	-9	12	6	caudate nucleus
13	3,60	0.0002	15	15	3	caudate nucleus
**Predicted outcomes: remembered vs. forgotten (** **Fig. 5A** **)**						
6	3,57	0.0002	15	42	-9	vmPFC
**Predicted vs. unpredicted outcomes, inclusively masked by s.m. of predicted outcomes (** **Fig. 5B** **)**						
23	3,70	0.0001	0	45	-15	vmPFC
**Predicted outcomes: reward vs. no reward (** **Fig. 5C** **)**						
184	5,73	0.00000002	-9	39	-12	vmPFC
	5,19	0.0000002	6	42	-6	vmPFC
17	4,36	0.000009	-6	15	-6	nucleus accumbens
	3,49	0.0003	12	21	-6	caudate nucleus
23	3,80	0.00009	-24	-36	0	hippocampus
	3,79	0.00009	-33	-39	3	hippocampus
**Outcomes: 'predictability' × 'reward' interaction (** **Fig. 5D** **)**						
9	3,31	0.00054	-27	-36	0	hippocampus
**Predicted rewarding outcomes: remembered vs. forgotten (** **Fig. 5E** **)**						
7	3,79	0.00009	12	42	-12	vmPFC
**Predicted rewarding outcomes vs. reward-predicting cues (** **Fig. 6** **)**						
232	5,62	0.00000003	12	36	-3	vmPFC
	5,52	0.00000005	-6	42	-15	vmPFC
	4,05	0.00003	-15	42	6	medial PFC
12	5,02	0.0000005	9	21	-3	caudate nucleus
33	4,37	0.000009	24	-39	-6	parahippocampal gyrus
	3,65	0.0002	33	-45	3	right ventricle
	3,25	0.0007	33	-39	-3	hippocampus
11	3,91	0.00006	-21	-36	0	hippocampus

Next, we analyzed changes in BOLD responses associated with the effect of reward prediction on memory. As described above, reward-predicting cues in predictable blocks were better remembered than cues in unpredictable blocks which were followed by an unpredicted reward. To investigate the neural processes underlying this enhanced memory, we contrasted subsequently remembered and forgotten trials in these two conditions. In predictable blocks, subsequently remembered as compared to forgotten cues were associated with significantly increased activity in the right parahippocampal gyrus (peak MNI coordinates: 21, -51, -6 and 27, -48, -12; Fig. 3B). Within unpredictable blocks, in contrast, we did not find any subsequent memory effects in medial temporal regions. Moreover, the interaction of subsequent memory and predictability indeed revealed increased subsequent memory effects in the parahippocampal cortex for predictable cues (18, -48, -9; Fig. 3C). The enhanced memory for cues in predictable as compared to unpredictable blocks may thus be explained by the fact that only cues in predictable blocks recruit medial temporal regions during memory formation.

#### Outcomes

**Figure 4:**
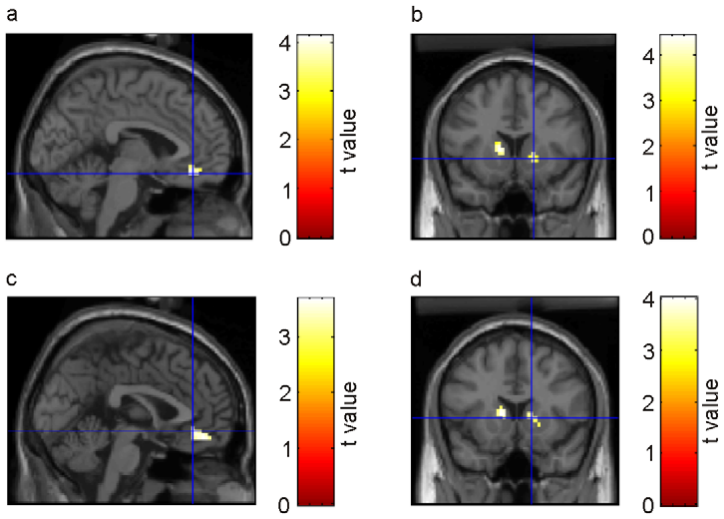
Activation of ventral striatum and ventromedial prefrontal cortex during unpredicted and predicted rewards and outcomes. (a) Increased BOLD response in vmPFC for predicted vs. unpredicted rewards. (b) Reverse contrast was associated with activation of the ventral striatum. (c) Increased BOLD response in vmPFC for predicted vs. unpredicted outcomes (rewards and no rewards). (d) Again, the reverse contrast activated the ventral striatum.

**Figure 5:**
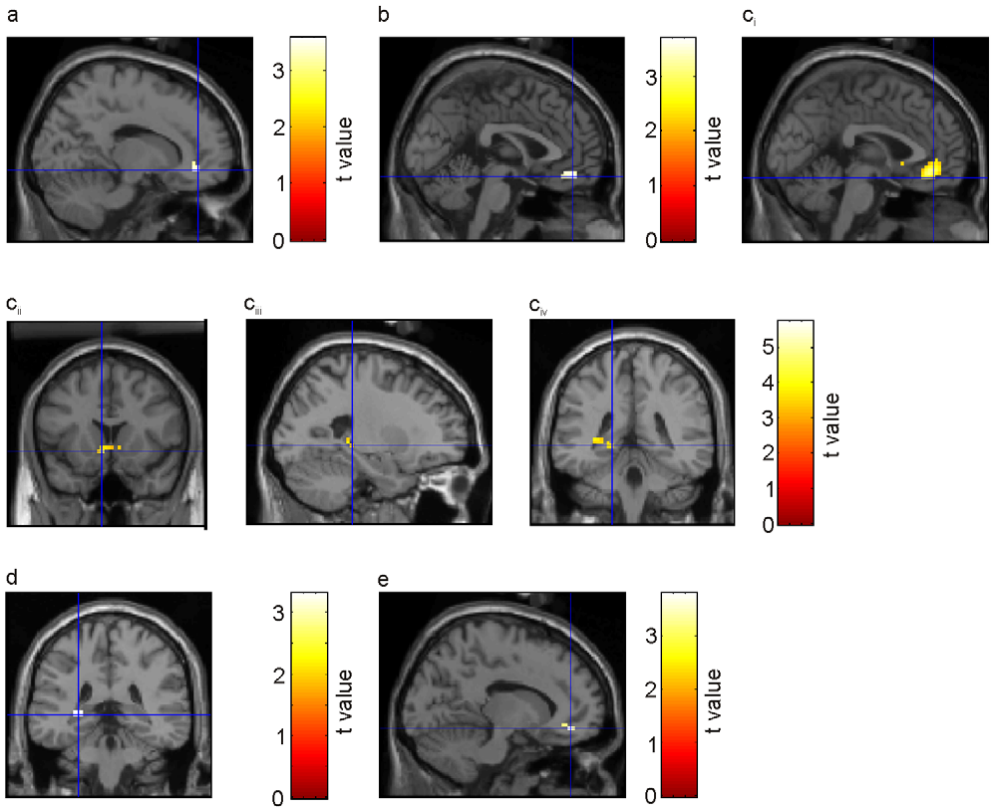
Subsequent memory effects and activation of memory-related regions during reward consumption. (a) Increased BOLD response in vmPFC for remembered contrasted to forgotten predicted outcomes. (b) Contrast of predicted vs. unpredicted outcomes masked (inclusively) by the contrast of forgotten vs. remembered outcomes. (c) Increased BOLD response for rewarding compared to non rewarding outcomes in predictable blocks in vmPFC (ci), nucleus accumbens (cii), and hippocampus (ciii and civ). (d) Interaction of reward and predictability for outcomes associated with activation of the posterior hippocampus. (e) Increased BOLD response in vmPFC for remembered contrasted to forgotten predicted rewards.

We first tested whether we could replicate previous findings that unpredicted rewards activated striatal regions, while predictable rewards induced activity in the vmPFC [3], [4]. Indeed, the contrast of predicted vs. unpredicted rewards was associated with significant activity in the medial orbitofrontal cortex (-3, 39, -15; Fig. 4A), while the reverse contrast showed significant activity in bilateral ventral striatum (left caudate nucleus, -12, 12, 6; and a cluster centered in the internal capsule between caudate, nucleus accumbens and putamen, 15, 9, 0; Fig. 4B). Similar effects were observed if all (rewarding and non-rewarding) outcomes were taken into account: Predicted outcomes activated the medial orbitofrontal cortex to a stronger degree (0, 45, -15; Fig. 4C), while unpredicted outcomes activated the bilateral ventral caudate nucleus (-9, 12, 6, and 15, 15, 3; Fig. 4D). A subsequent memory analysis for the predicted outcomes showed increased BOLD responses in the vmPFC (15, 42, -9; Fig. 5A). In the unpredictable blocks, in contrast, remembered outcomes did not induce any significant additional activation in comparison to forgotten ones. To verify whether the same vmPFC regions related to presentation of predicted outcomes are also associated with memory formation of these items, we inclusively masked the contrast of predicted vs. unpredicted outcomes with the subsequent memory contrast for predicted outcomes (at a masking threshold of p<0.05). The results are presented in Fig. 5B: Indeed, this contrast revealed a significant activation in the ventromedial wall of the prefrontal cortex (0, 45, -15).

As described in the Introduction, we were particularly interested in activity during consumption of predicted and unpredicted rewards. In predictable blocks, rewarding as compared to non-rewarding outcomes were associated with increased activation in bilateral vmPFC (-9, 39, -12; and 6, 42, -6), bilateral ventral striatum (left nucleus accumbens, -6, 15, -6; right caudate, 12, 21, -6), and in the bilateral hippocampus (-24, -36, 0; and -33, -39, 3; Fig. 5C). The striatal regions were not observed in the corresponding contrast for unpredictable blocks; the interaction of ‘reward’ × ‘predictability’ showed increased activity in the left posterior hippocampus for predictable rewards (-27, -36, 0; Fig. 5D). Finally, for rewarding outcomes within predictable blocks, we found a subsequent memory effect in the right vmPFC (12, 42, -12; Fig. 5E). For non-rewarding outcomes, in contrast, there was no significant activation. These results show that consumption of rewards was only activated with activation of memory-related regions in the medial temporal lobe if these rewards were predictable, but not if they occurred unpredictedly.

#### Cues vs. outcomes

**Figure 6:**
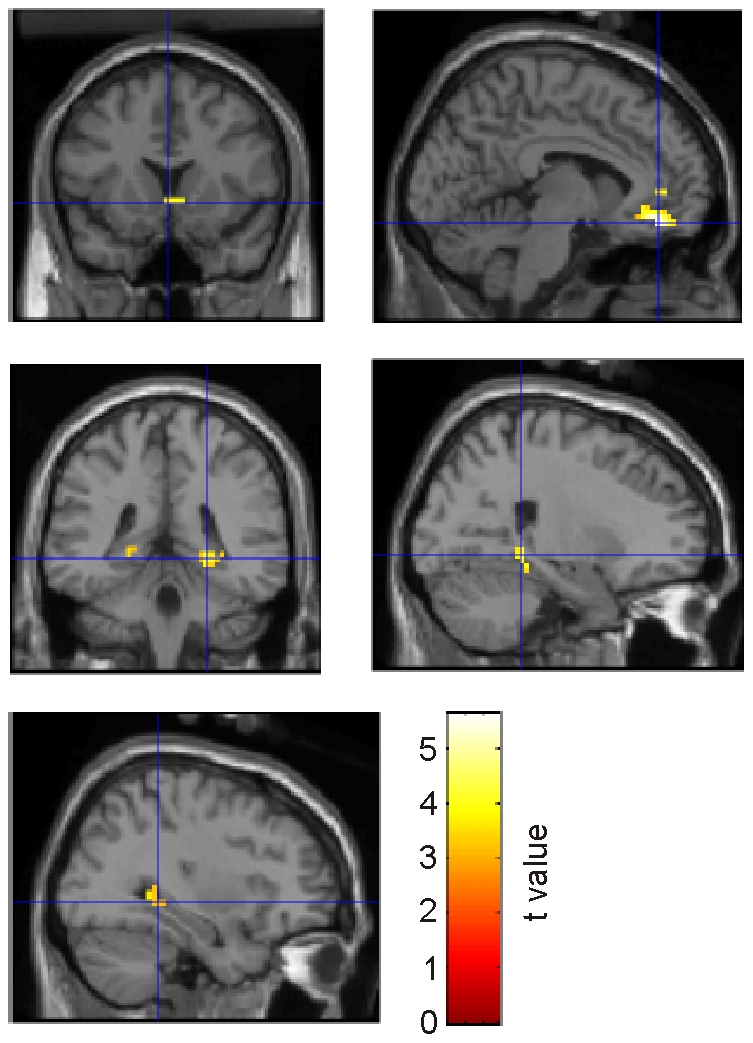
Reward consumption vs. anticipation. Increased BOLD response in vmPFC, bilateral ventral striatum, parahippocampal gyrus, and hippocampus during processing of predicted rewards vs. reward-predicting cues.

Our results described thus far show that rewarding as compared to non-rewarding outcomes are associated with activation of striatal regions even if they have been predicted. Next, we directly compared activity during reward anticipation and consumption. Interestingly, the contrast between predicted rewarding outcomes and reward-predicting cues revealed significant activity not only in various medial prefrontal regions (including ventromedial areas, e.g., -6, 42, -15), but also in the right caudate nucleus (right caudate, 9, 21, -3) and the bilateral hippocampus (33, -39, -3; and -21, -36, 0; Fig. 6). The reverse contrast revealed no significant activation within our search volume.

## Discussion

To summarize, our findings replicate previous results that reward-predicting cues and unpredicted rewards are associated with enhanced activity of the ventral striatum, while predictable rewards induce an increased BOLD response in the vmPFC. However, they move beyond previous studies by showing that (1) activation of the vmPFC associated with consumption of predicted rewards enhances memory formation, and that (2) activation of the ventral striatum facilitates memory formation only in a predictable situation.

### Experimental paradigm

We aimed at establishing a paradigm in which both the phase prior to reinforcement learning could be tested, when rewards were unpredicted, and the situation after learning, when cues reliably predicted a reward. It should be noted that the receipt of money is not a primary reward; however, on a neural level anticipation and receipt of monetary and taste rewards appears to elicit activation in very similar regions [5]. We made sure that the relationship between cues and outcomes was quickly learned in one type of blocks, while there was no such relationship in the other type of blocks. The blocks where participants could learn easily (A-type blocks) correspond (after the first three initial trials) to the situation ‘after learning’, while the blocks where learning was not possible (B-type blocks) correspond to the phase ‘before learning’. Behaviorally, this design yielded the expected results for the number comparison task: Within predictable blocks, we found that reward prediction reliably improved subjects’ performance in this task, as measured by both accuracy and RTs (Fig. 2). In fact, reaction times during non-rewarded trials in A-type blocks were considerably slower than for the unpredictably rewarded or not rewarded trials in B-type block (495 ms vs. 413 and 409 ms). This indicates that subjects indeed responded slower if they knew they would not be rewarded. Possibly, they did so because they implicitly exploited the variable reaction time threshold in order to maximize their outcome to the rewarded cues. However, in our experiment, reaction times only served to ensure that subjects actually had learned about reward contingencies, and the slow reaction times for un-rewarded trials in A-type block clearly indicate that subjects were aware that no reward would follow. In trials where stimulus category did not reliably predict a reward, in contrast, there was no effect of stimulus category on RTs and accuracy, which were both about half-way between rewarded and unrewarded trials in predictable blocks. These results indicate that reward expectation indeed influenced subjects' behavior in the number-comparison task in the expected direction. It is unlikely that the relatively poor recognition memory performance for the faces presented during outcome was due to the fact that subjects did not attend these faces, because subjects needed to indicate whether the presented person had an age of below or above 30 years.

### FMRI results: Activation of the ventral striatum

Consistent with previous studies on reward processing, we observed an increased BOLD response during unpredicted rewards in regions previously associated with reward processing: The BOLD contrast between unpredicted and predicted rewards was associated with significant activity in regions of the ventral striatum (Fig. 4B). This BOLD response is most likely due to phasic activation of dopaminergic midbrain neurons in response to reward consumption [17]. The ventral striatum was also increasingly activated by reward-predicting cues as compared to no reward predicting cues (Fig. 3A), consistent with previous results [12], [18].

It is still a debated question whether the ventral striatum is only activated during reward anticipation [3], [5], [19], or also when subjects are informed about the outcome [6], [7], [8], [20]. Our results are consistent with the latter findings, because we found increased bilateral ventral striatum activation not only for cues that predicted a reward (as compared to cues predicting no rewards; Fig. 3A), but also for predicted rewarding outcomes as compared to predicted non-rewarding outcomes (Fig. 5C). Interestingly, striatal response upon a predicted reward was even higher than on the predicting cue itself (Fig. 6). Thus, the ‘shift’ hypothesis could not be replicated in our study, because activity did not decrease but increased in outcome phases.

In principle, it would be interesting to investigate whether striatal activity is sustained throughout the anticipation period, i.e., whether it also occurs in the absence of the cue. However, this idea is difficult to test in our current dataset. First, the experimental power of the imaging data is limited by the fact that we already have 16 regressors to account for activity in a four-way design (item type, predictability, reward, memory). Using additional regressors to model activity during the late anticipation phase would further reduce experimental power. Second, interpretation of activity during the late anticipation phase is difficult because we present a movie of an opening door, which is associated with relatively complex visual processing. Therefore, contrasts involving this middle time period would probably be rather unreliable due to the highly variable activation patterns. Third, our predictions mainly concern memory effects based on phasic dopamine responses during cue presentation and outcome processing. Although more tonic dopamine effects are also observed throughout the anticipation period, we did not have similarly clear predictions for these effects on memory.

### FMRI results: Effects of memory

Overall, we found that cues were better remembered than outcomes. The pictorial cues consisted of consumer goods which might be more distinctive than the human faces used in our study (although this effect is probably not generalizable). Even more importantly, participants might have studied these pictures more intensively than the faces, because they were seeking the hidden rule (the category) behind them. Moreover, reward-predicting cues were significantly better remembered than cues followed by rewards in unpredictable blocks, and predicted outcomes were better remembered than unpredicted outcomes (Fig. 2). It should be noted that the differences between activation during processing of cues and outcomes might be influenced by the different stimulus categories. In particular, activation of the ventromedial prefrontal/orbitofrontal cortex has been observed in various studies using faces as stimuli (for a review, see [21]). On the other hand, activation of this region was also found in rodents during reward processing when other stimuli than faces were used (e.g., [22], [23]); furthermore, a recent meta-analysis showed that the ventromedial prefrontal cortex was activated by emotional stimuli regardless of stimulus category (faces or scenes [24]). Enhanced memory for reward-predicting cues as compared to cues followed by rewards in unpredictable blocks may be explained by the fact that they activated the ventral striatum (Fig. 3A), which was previously found to facilitate memory formation [12] and was accompanied by medial temporal activation for subsequently remembered reward-predicting cues (Fig. 3B). In our main analysis where we collapsed across “sure” and “unsure” responses, we did not find a significant effect of “reward” on subsequent memory for cues in predictable blocks. At first sight, this result appears to be inconsistent with the findings of Wittmann et al. [12] and Adcock et al. [13]. However, in those two studies reward-predicting cues were only associated with better memory if tested 24 hours [13] or two weeks [12] later, but not if tested shortly after encoding (this was tested as well only in the study by Wittmann and coworkers). Therefore, our results lend further support to the idea that reward-predicting cues facilitate memory consolidation and, on a neurophysiological level, act on the late phases of long-term potentiation. Interestingly, however, we did find an effect of reward on memory tested briefly afterwards when only “sure old” responses were considered. Due to insufficient trial numbers, we could not analyze the neural signature underlying this effect. It might be related, however, to a specific effect of reward on recollection as compared to familiarity: According to the dual-process theory of recognition memory, these two processes are independent [15]. This distinction of labor on a psychological level appears to be paralleled by a double dissociation between sub-regions of the medial temporal lobe supporting recollection and familiarity [25]. According to this view, whereas the hippocampus proper is necessary for recollection [26], the surrounding regions including the ento- and perirhinal cortex support recognition based on familiarity. “Sure old” responses are probably mostly based on recollection, whereas “unsure old” responses probably rather depend on familiarity (or guesses) [27]. Previous fMRI studies showed that experimental conditions activating the dopaminergic midbrain specifically supported conscious recollection [12], [28]. Therefore, the significant effect of reward on memory measured by “sure old” responses might be due to this specific effect on recollection.

For the outcomes, activation of the ventral striatum alone cannot explain effects on memory, because predicted outcomes activated the ventral striatum to a lesser degree than unpredicted outcomes (Fig. 4D), but were better remembered than the latter (Fig. 2). Here, activation of the vmPFC appears to play an important role: As described above, additional activation of this region was observed during processing of predicted outcomes (Fig. 4C). Indeed, we found that specifically those predicted outcomes which were subsequently remembered activated the vmPFC (Fig. 5A). This finding appears to contrast with studies in rodents, which indicate that the vmPFC is crucial for learning from unexpected outcomes (e.g., [22], [23]). However, these studies did not investigate memory for items which were directly associated with unpredicted outcomes, but rather the consequences of unexpected outcomes for subsequent behavior. Activation of the vmPFC has been previously associated with enhanced subsequent memory (e.g., [29], [30]), possibly due to its massive connections to the medial temporal lobe [31], [32]. Indeed, a previous fMRI study revealed increased functional connectivity between vmPFC and medial temporal lobe during memory formation [33]. Our finding of increased BOLD responses both in vmPFC and various medial temporal regions for predicted rewards as compared to predicted non-rewarding outcomes (Fig. 5C) are consistent with a functional role of these connections for memory formation of items which are directly associated with reward consumption. Interestingly, Tsukiura and Cabeza [28] recently showed that joint activation of vmPFC and hippocampus facilitated memory for smiling as compared to neutral faces; as perception of a smiling face can be considered a rewarding event [34], the results of this study directly support our hypothesis.

#### Conclusions

To conclude, we found that the connection between reward processing, striatal areas and memory formation within the MTL appears to occur only in a predictable situation. For cues, we found (as expected) that reward and predictability have a major influence on their subsequent memory, and that striatal as well as medial temporal regions play an important role in these incidents. These data are consistent with the hypothesis that activation of regions receiving dopaminergic inputs enhances hippocampus-dependent memory, e.g. [8]. In contrast, predicted outcomes were better memorized than unpredicted ones, though it were the unpredicted ones that enhanced activity in the striatum, while the predicted ones activated the vmPFC. In this case, memory formation was thus associated with activity in vmPFC. From an evolutionary perspective, individuals have to acquire knowledge about achievable gratifications when they can reach them by modifiable behavior. Therefore it makes sense that an expected reward is memorized as well at the first hints to it. Subjects learn to adapt to their surroundings and to gain benefits, but unpredicted rewards do not help to adapt to future events and to adjust behavior; learning and memorizing make sense only in a logically structured environment.
